# Differentiation of human embryonic stem cells into corneal epithelial progenitor cells under defined conditions

**DOI:** 10.1371/journal.pone.0183303

**Published:** 2017-08-15

**Authors:** Canwei Zhang, Liqun Du, Kunpeng Pang, Xinyi Wu

**Affiliations:** 1 Department of Ophthalmology, Qilu Hospital of Shandong University, Jinan, Shandong, PR China; 2 The Key Laboratory of Cardiovascular Remodeling and Function Research, Chinese Ministry of Education and Chinese Ministry of Health, Qilu Hospital of Shandong University, Jinan, Shandong, PR China; Xiamen University, CHINA

## Abstract

The development of cell-based therapies using stem cells represents a significant breakthrough in the treatment of limbal stem cell deficiency (LSCD). The aim of this study was to develop a novel protocol to differentiate human embryonic stem cells (hESCs) into corneal epithelial progenitor cells (CEPCs), with similar features to primary cultured human limbal stem cells (LSCs), using a medium composed of DMEM/F12 and defined keratinocyte serum-free medium (KSFM) (1:1) under different carbon dioxide (CO_2_) levels in culture. The differentiated cells exhibited a similar morphology to limbal stem cells under 5%, 7%, and 9% CO_2_ and expressed the LSC markers ABCG-2 and p63; however, CK14 was only expressed in the cells cultured under 7% and 9% CO_2_. Quantitative reverse-transcription polymerase chain reaction (RT-PCR) analysis indicated that the ABCG2, p63, and CK14 levels in the 7% CO_2_ and 9% CO_2_ groups were higher than those in the 5% CO_2_ group and in undifferentiated hESCs (p<0.05). The highest expression of ABCG2 and p63 was exhibited in the cells cultured under 7% CO_2_ at day 6 of differentiation. Western blotting indicated that the ABCG2 and p63 levels were higher at day 6 than the other time points in the 7% CO_2_ and 9% CO_2_ groups. The highest protein expression of ABCG2 and p63 was identified in the 7% CO_2_ group. The neural cell-specific marker tubulin β3 and the epidermal marker K1/10 were also detected in the differentiated cells via immunofluorescent staining; thus, cell sorting was performed via fluorescence-activated cell sorting (FACS), and ABCG2-positive cells were isolated as CEPCs. The sorted cells formed three to four layers of epithelioid cells by airlifting culture and expressed ABCG2, p63, CK14, and CK3. In conclusion, the novel induction system conditioned by 7% CO_2_ in this study may be an effective and feasible method for CEPC differentiation.

## Introduction

Corneal epithelium is continuously renewed by the proliferation and differentiation of stem cells located in the basal layer of the limbus, and it plays an important role in maintaining a clear, healthy cornea and preserving vision [1,2]. Destruction or damage to limbal stem cells (LSCs) may cause limbal stem cell deficiency (LSCD) and the consequent absence of an intact epithelial layer, in addition to conjunctival ingrowth, neovascularization, chronic inflammation, impaired vision, and ultimately blindness [3,4]. Currently, cultured limbal epithelium transplantation has presented very encouraging clinical results for LSCD treatment [5,6]. However, there are several limitations in the source of limbal tissues [7]. Moreover, the risks of LSCD development in the donor eye was also a controversial issue for the transplantation of autologous limbal epithelium[8]. The development of cell-based therapies using stem cells represents a significant breakthrough in the treatment of LSCD, thus providing a more rational, less invasive, and better physiological treatment option in regenerative medicine for the ocular surface [9].

Human embryonic stem cells (hESCs) possess the features of unlimited proliferation paired with an ability to differentiate into cells of all three embryonic germ layers [10,11]. Recently, hESCs have demonstrated their clinical value. They have substantial potential in cell replacement therapy and regenerative medicine [12,13].

Previous studies have indicated that hypercapnia may improve the conservation and proliferation of hematopoietic progenitors [14,15]. Culture in a 10% carbon dioxide (CO_2_) environment results in a substantial enhancement in hamster eight-cell embryo development [16]. An enhanced differentiation particularly toward the mesodermal and endodermal lineages at cultures maintained and differentiated at lowered CO_2_ levels has also been reported [17]. This finding indicates that changes in the CO_2_ concentration for cell cultures may affect the growth and differentiation of stem cells. In our preliminary experiment, we determined that 7% CO_2_ has beneficial effects on the differentiation of corneal epithelial progenitor cells (CEPCs) from hESCs. Therefore, in this study, three CO_2_ concentrations (5%, 7%, and 9%) were selected to evaluate the differentiation efficiencies of CEPCs from hESCs. Collagen IV is a major basement membrane component of limbal and corneal epithelia [18,19]. Previous studies have shown that collagen IV may be used to differentiate mouse ESCs into CEPCs and provide a good substrate for the induction of LSCs from hESCs [20–22].

Differentiation of hESCs/induced pluripotent stem (iPS) cells into corneal epithelial cells or stem cells continues to pose a challenge because the growth factors and three-dimensional signals that control hESC differentiation have remained elusive [23]. Most previously published studies have relied on the use of undefined factors such as conditioned medium, PA6 feeder cells, Bowman’s membrane, or amniotic membrane [20,21,24,25]. Recently, several studies have focused on the differentiation of corneal epithelial cells from iPS cells under defined conditions, such as differentiation medium [26], small molecule inhibitors (SB-505124 and IWP-2) in combination with FGF [27], or collagen IV together with keratinocyte culture medium [28]. The use of defined differentiation conditions, free from animal-derived components, would minimize the potential risk of animal pathogen transmission, immune reactions, and graft rejection. A defined condition would also improve the repeatability and consistency of differentiation [27]. However, the defined conditions to differentiate hESCs into CEPC are not available.

In this study, we developed a novel protocol to differentiate hESCs into CEPCs using collagen IV and a differentiation medium composed of DMEM/F12 and defined keratinocyte-serum free medium (KSFM) under 7% CO_2_. This method has substantial potential to provide an infinite cell source of CEPCs, with similar phenotypic and functional characterization to LSCs, for basic biological research and LSCD treatment.

## Materials and methods

### Isolation and culture of hLSCs from human limbus tissue

LSCs were isolated from donor limbal rim, which was not suitable for transplantation, obtained from Qilu Hospital of Shandong University. All tissue collection complied with the guidelines of the Helsinki Declaration and was approved by the Medical Ethics Committee of Qilu Hospital of Shandong University, China. Written informed consent was obtained from the donors or next of kin. LSCs were isolated and cultured as previously described [3]. Primary cells were seeded on plates coated with 2% growth factor reduced Matrigel (354230, BD Biosciences, San Jose, CA, USA). The culture medium (LM) consisted of DMEM/F12 and DMEM (1:1) with 1% penicillin-streptomycin, 10% fetal bovine serum, 10 ng/ml EGF, 5 mg/ml insulin, 0.4 μg/ml hydrocortisone, 10^−10^ M cholera toxin, and 2×10^−9^ M 3,3’,5-triiodo-L-thyronine.

### Culture of human embryonic stem cells

The hESC line employed was H1 (WiCell Research Institute Inc., Madison, WI, USA), which was grown on hES-qualified Matrigel (Corning®, Tewksbury, MA, USA) coated plates with mTeSR1 medium (STEMCELL Technologies, Vancouver, Canada) as previously described [29,30]. The cells were incubated at 37°C in a humidified atmosphere that contained 5% CO_2_ and were passaged every 5 days.

### Differentiation of corneal epithelial progenitor cells

hESCs were released from the culture using 1 mg/ml dispase and plated into six-well tissue culture plates coated with collagen IV. The cells were cultured in mTeSR1 for 2 days and subsequently transferred into three different incubators, in which the CO_2_ concentration was 5%, 7%, and 9%, respectively; thus, the experimental groups were designated as 5% CO_2_, 7% CO_2_ and 9% CO_2_. The medium was changed into KSFM-DMEM/F12 (KDM). The hESCs grew slowly on Collagen IV-coated plates in mTeSR1, and clone detachment appeared after 3 days of culture; thus, hESCs cultured on hES-qualified Matrigel coated plates with mTeSR1 were used as the controls. The medium was replaced every day.

### Cell number counting

Cell number counting was performed to assess cell proliferation as previously described [31]. Briefly, hESCs were released from the culture and dispersed by gently pipetting up and down several times. The cells were then equally distributed into three groups (5% CO_2_, 7% CO_2_, and 9% CO_2_), with approximately 20 cell aggregates per well of six-well plates. At days 3, 6, 9, and 12 of differentiation, the cells were detached from the culture dishes using a 0.05% trypsin and 0.5 mM EDTA solution, and 0.4% (w/v) Trypan blue solution (Solarbio, China) was subsequently added to the cell suspension. The cells excluding the dye were considered viable and counted on a hemocytometer under optical microscopy.

### Immunofluorescent staining of cell cultures

Cells were fixed with 4% paraformaldehyde for 20 minutes and incubated in 0.5% Triton X-100 for 10 minutes and 5% goat serum for 30 minutes. The primary antibodies included mouse anti-ABCG2 (1:100; Abcam Technologies, Cambridge, UK), rabbit anti-p63 (1:100; Abcam Technologies, Cambridge, UK), rabbit anti-CK14 (1:100; Abcam Technologies, Cambridge, UK), mouse anti-CK3 (1:100; Santa Cruz Biotechnology, Santa Cruz, CA, USA), rabbit anti-Tubulin β3 (1:100; BioLegend, San Diego, CA, USA), mouse anti-CK1/10 (1:100; Santa Cruz Biotechnology, Santa Cruz, CA, USA) and rabbit anti-MITF (1:50; Sigma-Aldrich Corporation, St. Louis, MO). Cells were incubated with the primary antibodies overnight at 4°C. Secondary antibodies (1:100; all obtained from Beijing Zhongshan Technologies, Beijing, China) coupled to fluorescein isothiocyanate (FITC) or tetramethyl rhodamine isothiocyanate (TRITC) were subsequently applied for detection. The cells were then stained with 4, 6-diamidino-2-phenylindole (DAPI) to visualize the nuclei. Fluorescence was observed using a fluorescence microscope (model BH2RFL-T3, Olympus Corporation, Tokyo, Japan).

### Quantitative reverse-transcription polymerase chain reaction (qRT-PCR)

Total RNA was extracted from the differentiated cells with TRIzol reagent (Invitrogen Corporation, Carlsbad, CA, USA), and cDNA was synthesized with a First Strand cDNA Synthesis Kit (Toyobo, Osaka, Japan) according to the manufacturer’s protocol. Then, qRT-PCR was performed in triplicate on a sequence detection system (ABI Prism 7000; Life Technologies/Applied Biosystems, Inc., Foster City, CA, USA). The mean CT values were calculated, and the relative expression values were calculated from the delta CT values using the formula: 2^-△△CT^. The volume of RT-PCR was 20 μl, including 2 μl cDNA, 10 μl SYBR Green Real-time PCR Master Mix (Toyobo, Osaka, Japan), 1 μl each of specific forward and reverse primers, and 6 μl sterile water. Quantitative RT-PCRs were run in duplicate using a LightCycler (Roche, Basel, Switzerland) at 95°C for 30 seconds, followed by 40 cycles of 95°C for 5 seconds, 56–60°C for 10 seconds, and 72°C for 60 seconds. The primers used were as follows: ABCG2, forward 5’- ACGAACGGATTAACAGGGTCA- 3’; and reverse 5’- CTCCAGACACACCACGGAT- 3’; P63a, forward 5’- TGCCCAGACTCAATTTAGTGA- 3’; and reverse 5’- GAGGAGCCGTTCTGAATCTG- 3’; CK14, forward 5’- TCCTTCGCACCAAGAACTGAG- 3’; and reverse 5’- CAGGAGAGGGGATCTTCCAGT- 3’; CK3, forward 5’- GGATGTGGACAGTGCCTATATG- 3’; and reverse 5’- AGCGTCGTAGAGGGTCCTT- 3’; OCT4, forward 5’- CAAAGCAGAAACCCTCGTGC- 3’; and reverse 5’- TCTCACTCGGTTCTCGATACTG- 3’; GAPDH, forward 5’- TGAACGGGAAGCTCACTGG- 3’; and reverse 5’- TCCACCACCCTGTTGCTGTA- 3’. For the normalization of the gene expression levels, the gene-to-GAPDH (housekeeping gene) was calculated and compared to that of the undifferentiated hESCs. LightCycler software and LightCycler Relative Quantification software were used to analyze the data.

### Western blot analysis

Western blotting proceeded as previously described [32]. Briefly, cells were collected and lysed by shaking at 4°C for 30 minutes in RIPA buffer that contained protease inhibitors. The cell lysates were centrifuged at 12,000 g for 15 minutes at 4°C. The supernatant was boiled for 10 minutes. The total protein was quantified, and the protein samples were subjected to 10% sodium dodecyl sulfate-polyacrylamide gel electrophoresis (SDS-PAGE) and subsequently transferred to nitrocellulose membranes. The membranes were blocked with 5% skim milk in Tris-buffered saline for 2 hours at room temperature prior to overnight incubation at 4°C with primary antibodies, washed with Tris-buffered saline and incubated with secondary antibodies for 2 hours at 37°C. Protein bands were visualized using enhanced chemiluminescence as described by the supplier. A densitometric analysis was conducted with Quantity One software (Bio-Rad Laboratories, Berkeley, CA, USA).

### Fluorescence-activated cell sorting (FACS) analysis and purification

The cell sorting analysis and purification were performed as previously reported [23,33]. The differentiated cells were digested using trypLE™ Express (Gibco, Thermo Fisher Scientific, Waltham, MA, USA) for 10 minutes at 37°C and then incubated with PerCP-Cy™5.5 Mouse anti-ABCG2 antibody (1:20, BD Technologies, Durham, NC, USA) in complete DMEM/F12 medium for 30 minutes at 4°C. The cells were washed three times with ice-cold phosphate buffered saline (PBS) prior to analysis using a Guava® easyCyte^TM^ cytometer (Millipore Technologies, Billerica, MA, USA). The negative controls included isotype-matched irrelevant antibodies. Cell isolation was conducted on a FACS Aria II (BD Biosciences, San Jose, CA, USA).

### Airlifting culture

CEPCs were gently seeded at 1.5 × 10^4^ mm^-2^ on the Bowman’s membrane side of acellular porcine limbal matrix (APLM) prepared as previously described [34–36]. The cells were cultured in a submerged condition for 7 days and subsequently changed to air-lift culture for an additional 7 days to induce epithelial stratification [20]. The medium was LM and changed daily during airlifting.

### Histology

The epithelial sheets were fixed and processed, embedded into paraffin blocks and mounted as 4-μm-thick paraffin-embedded sections. Hematoxylin and eosin (HE) staining was performed using conventional methods. For immunohistochemical analyses, deparaffinized sections were treated with a primary antibody at 4°C overnight and subsequently incubated with secondary antibodies conjugated with FITC or TRITC in the dark for 1 hour at room temperature. The primary and secondary antibodies were the same as those used in immunocytochemistry. Fluorescence was observed under a fluorescence microscope. For western blotting and qRT-PCR, the cell sheets were snap frozen in liquid nitrogen and grinded to powder using a precooled pestle; hESCs seeded on APLM were used as the control. The subsequent steps were similar to those of the previously described cells.

### Statistical analysis

All statistical analyses were performed using SPSS 13.0. Data are presented as the mean ± standard deviation. The t-test and One-way analysis of variance (ANOVA) were used for statistical analysis. Differences were considered statistically significant at p < 0.05.

## Results

### Isolation, culture, and identification of hLSCs

**Fig 1:**
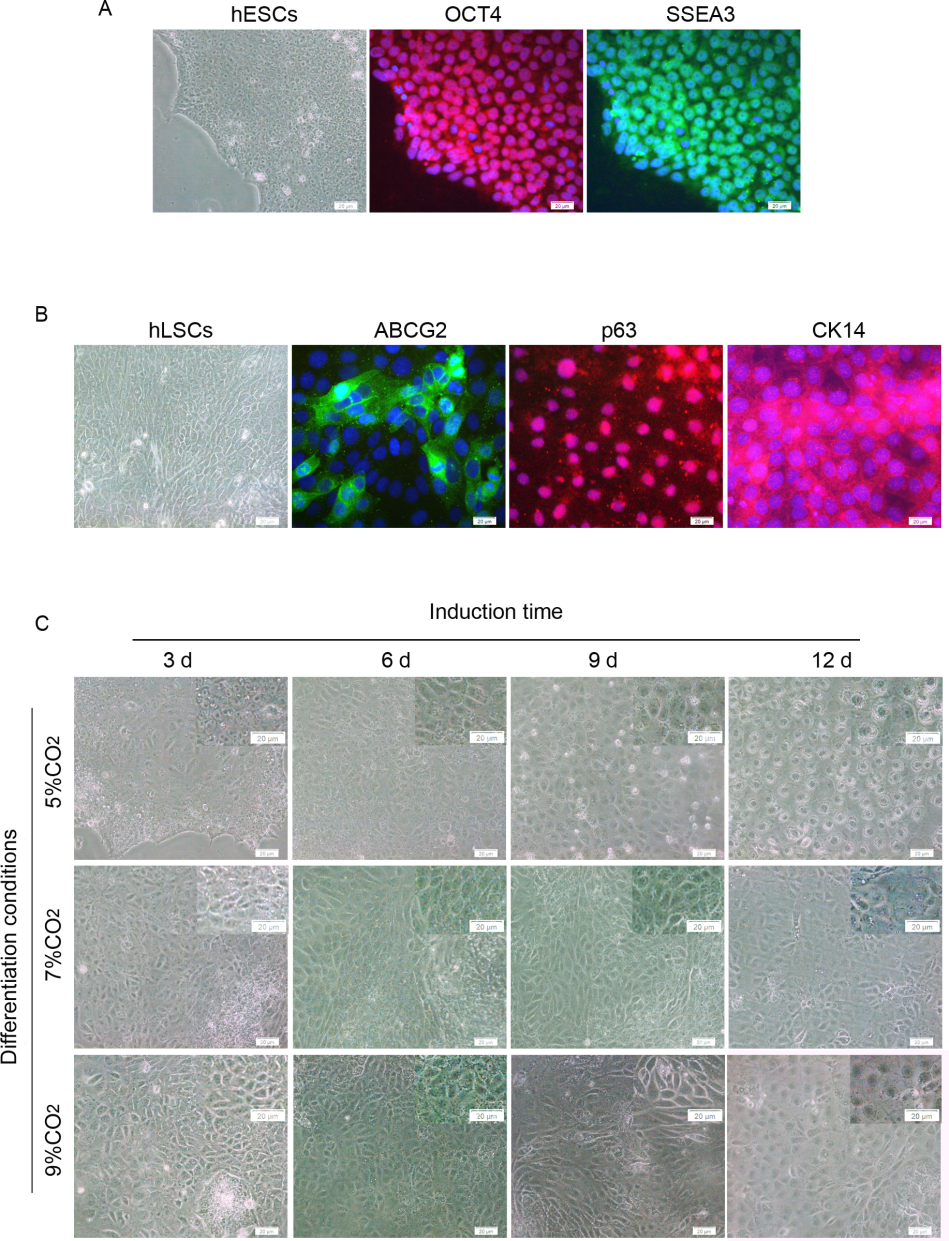
Primary cultured hLSCs and morphology of cells from hESCs at different stages of differentiation. (A) Microscopic image of hESCs and the expression of OCT4 and SSEA-3. (B) Morphology of hLSCs and immunofluorescent staining of ABCG2, p63, and CK14. (C) Representative images of the differentiated cells at days 3, 6, 9, and 12 of differentiation. Scale bar, 20 μm.

Human LSCs were successfully isolated from human limbal tissue and cultured in feeder-free cell culture conditions as previously reported [2]. The cells were small and cuboidal and exhibited the expression of the LSC markers ABCG2, p63, and CK14, similar to previous reports [2,37] (Fig 1B).

### Morphological changes and proliferation capacity of differentiated hESCs

**Fig 2:**
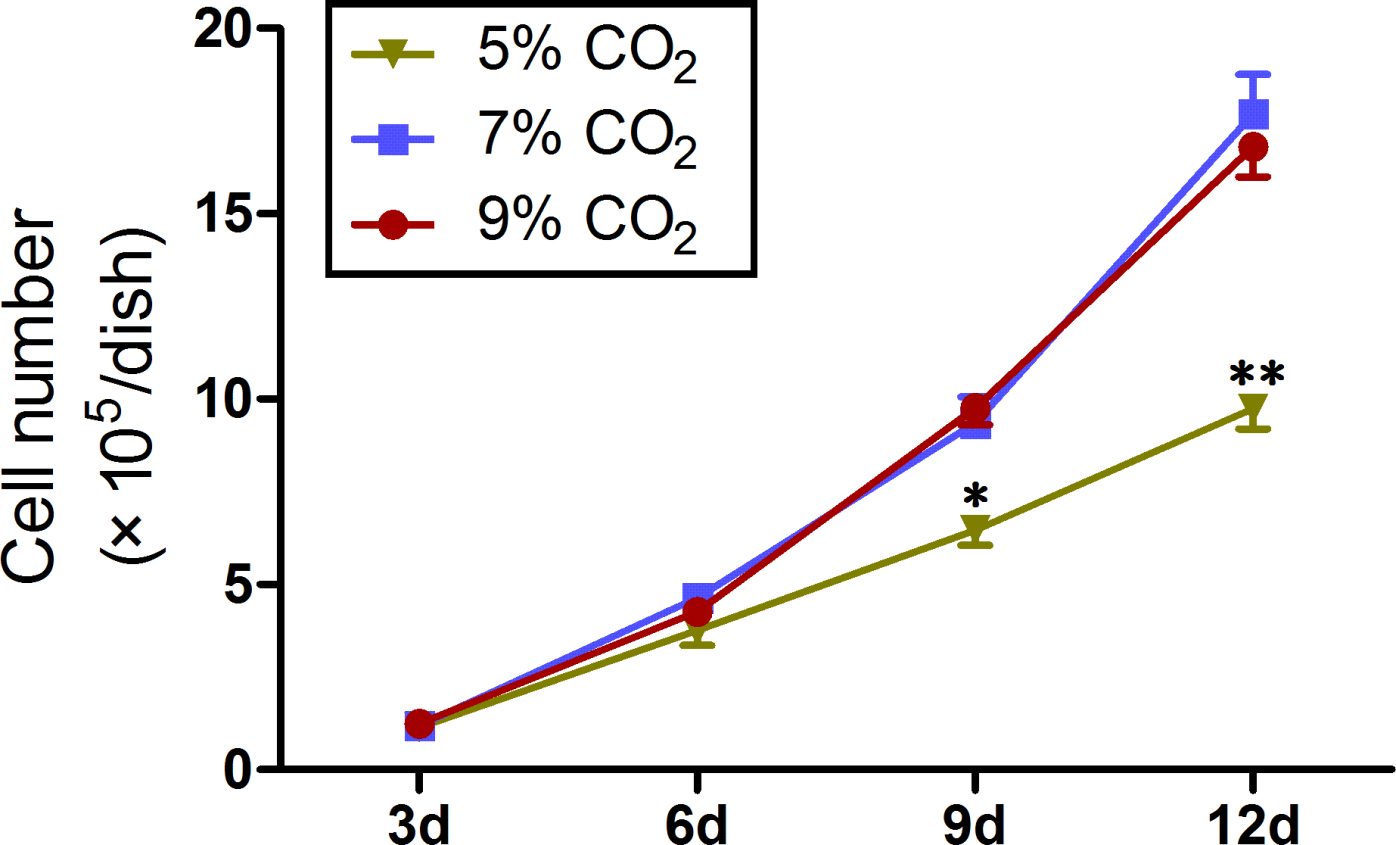
Number of cells counted using a hemocytometer. Data represent mean ± SD of three independent experiments with triplicate dishes.

Within the first 3 days of differentiation, the cells became relatively flatter and spread out appearing under 7% CO_2_. On day 6, the majority of the cells changed into small cuboid cells and formed regularly arranged, cobble stone-like cell sheets, similar to primary cultured LSCs. On day 9, the number of cells increased, and nearly all cells exhibited a cobble stone-like morphology. On day 12, the cells became much flatter, and the cells with an increased cell size appeared in the periphery (Fig 1C). A similar morphological change was identified in the cells cultured under 5% and 9% CO_2_; however, the cells under 5% CO_2_ were much flatter and grew more slowly than the cells cultured under 7% CO_2_ and 9% CO_2_ (Fig 1C). To define the cell growth in the three groups, cell number counting was performed. Trypan blue dyed cell counting showed that the cell density in the cultures under 5% CO_2_ was significantly lower than that in the cultures under 7% CO_2_ and 9% CO_2_ on days 9 and 12 (p < 0.05) (Fig 2).

### Differentiation of hESCs into CEPCs

**Fig 3:**
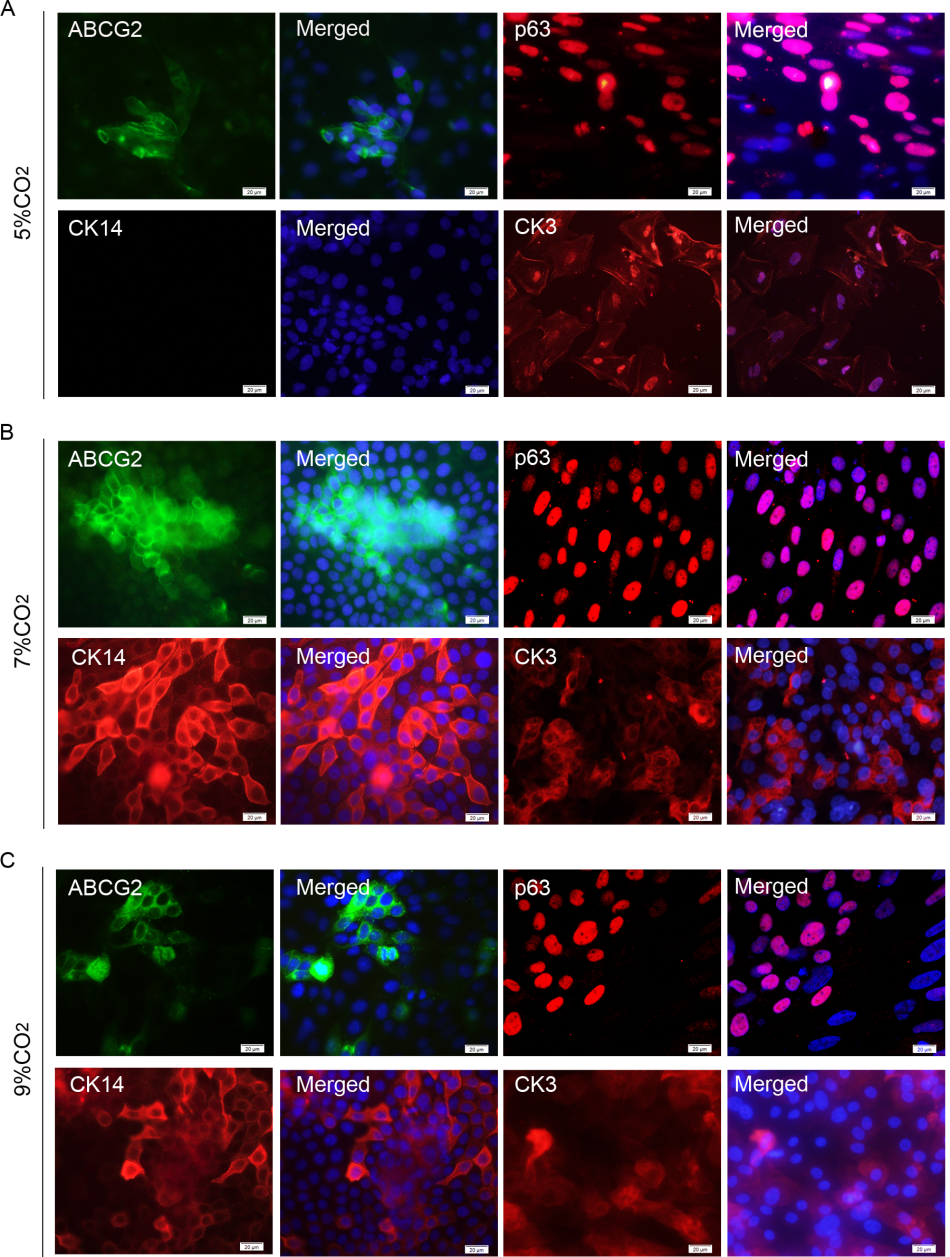
Immunofluorescence images of the differentiated cells. Expression of ABCG2, p63, and CK14 at day 6 and CK3 at day 12 of differentiation in the cells cultured under 5% CO_2_ (A), 7% CO_2_ (B), and 9% CO_2_ (C). Scale bar, 20 μm.

The differentiation of hESCs into CEPCs was confirmed via immunofluorescence, qRT-PCR analysis, and western blotting. The immunofluorescence of the fixed cells showed that the expression of ABCG2, p63, and CK14, which are the markers for LSCs [26,38,39], were strongly expressed in the cytomembrane, nucleus, and cytoplasm of the induced cells under 7% and 9% CO_2_, respectively. CK3, the terminally differentiated cell marker, which is specific to corneal epithelium [40,41], was also identified at the periphery of some colonies on day 12 of differentiation (Fig 3B and 3C). However, the immunofluorescence signals of CK14 could not be detected in the cells cultured under 5% CO_2_, and the expression of ABCG2 and CK3 was also very weak in these cells (Fig 3A). These data indicate that hESCs cultured on collagen IV in KSFM-DMEM/F12 may be differentiated into CEPCs, and a slightly elevated CO_2_ level is more conducive to the differentiation of CEPCs from hESCs. The expression of CK3 demonstrated that this culture condition may not be helpful in the maintenance of stem cell properties, and the cells at the periphery of some colonies can further differentiate into corneal epithelial cells.

**Fig 4:**
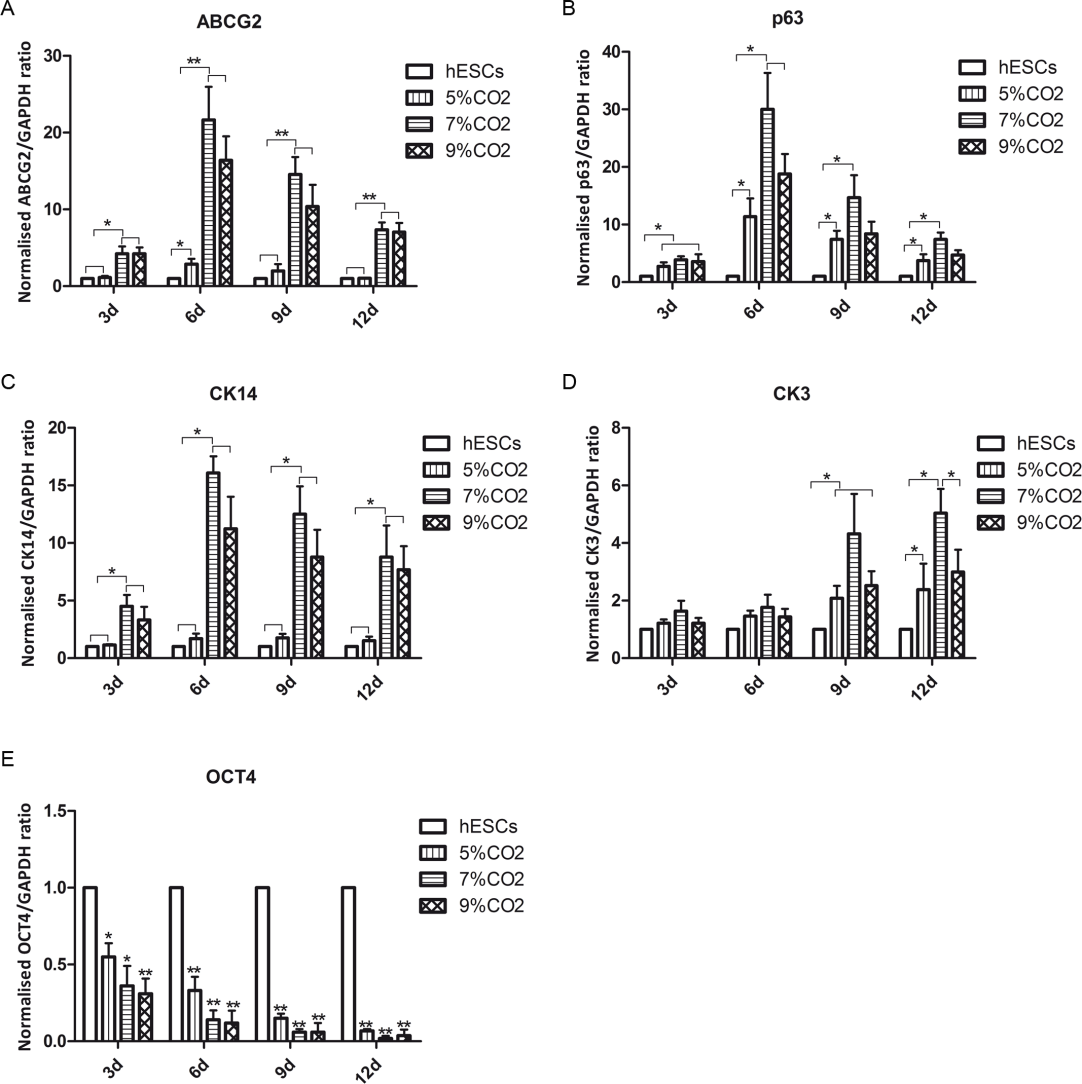
Time course of ABCG-2, p63, CK14, and CK3 during hESC differentiation culture (3, 6, 9, and 12 days). Data are representative of three experiments and presented as the mean ± SD.*p < 0.05, **p < 0.01.

Quantitative RT-PCR analysis was performed to confirm the differentiation of hESCs into CEPCs. In all three groups, the expression of p63 began to increase on day 3 (Fig 4B). In the 7% CO_2_ and 9% CO_2_ groups, but not in the 5% CO_2_ group, the expression of ABCG2 and CK14 exhibited a significant increase on day 3 (Fig 4A and 4C). The ABCG2, p63, and CK14 levels peaked at the 6-day time point in the 7% CO_2_ and 9% CO_2_ groups. The highest expression of ABCG2 (averaged 21.65-fold), p63 (averaged 30.01-fold), and CK14 (averaged 16.07-fold) was identified in the 7% CO_2_ group. However, we did not identify significant differences in the levels of ABCG2, p63, and CK14 between the 7% CO_2_ group and the 9% CO_2_ group. The expression of CK3 was elevated in all experimental groups on days 9 and 12 (Fig 4D). The upregulation of CK3 accompanied with decreased expression of ABCG2, p63, and CK14 decreased in the 7% CO_2_ and 9% CO_2_ groups, which suggests that corneal epithelial differentiation may be in progress. OCT4, a marker of undifferentiated hESCs, is crucial to the establishment of pluripotential identity [42]. In all experimental groups, the expression of OCT4 significantly decreased within 6 days of differentiation and declined to nearly negligible levels by day 12 (Fig 4E). These findings suggested that there was a loss of hESC pluripotency during the differentiation process.

**Fig 5:**
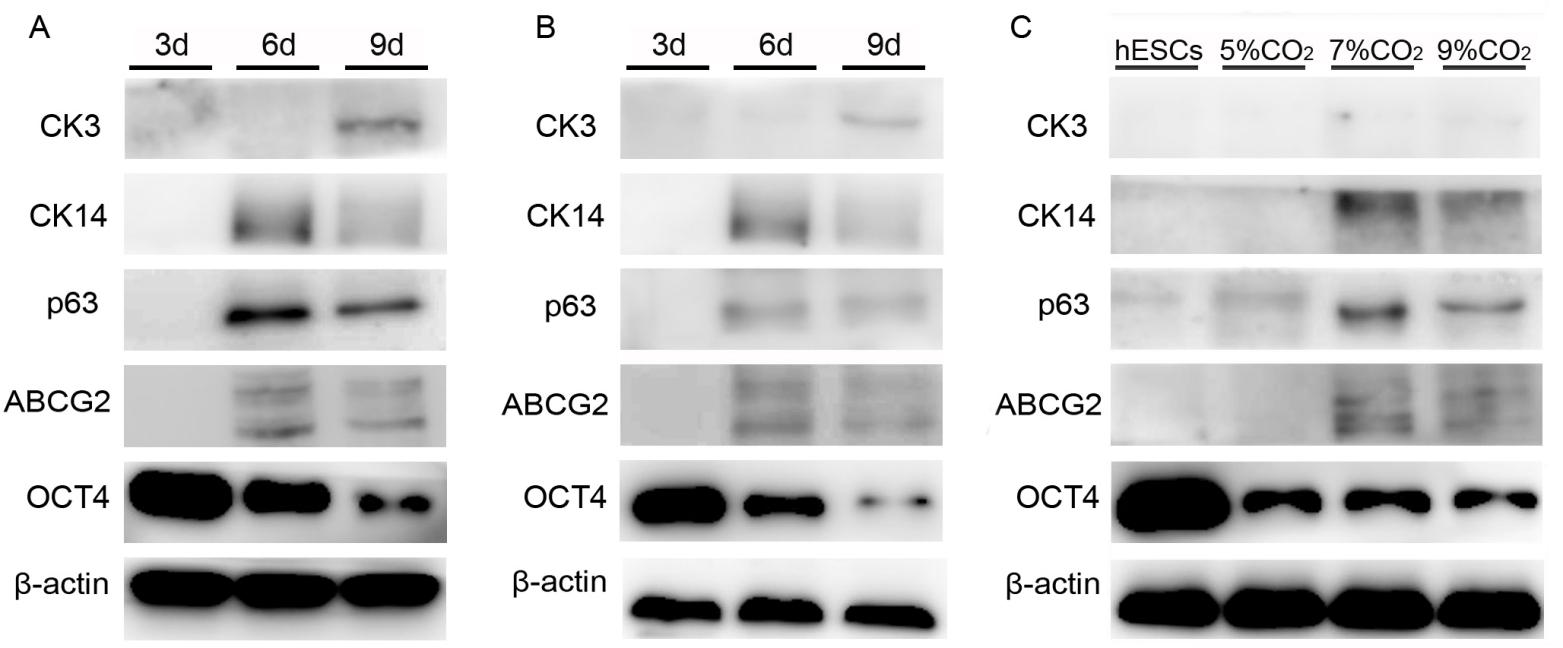
Protein expression of CEPC and hESC markers at different time points and in different culture conditions. (A-B) ABCG2, p63, CK14, CK3, and OCT4 protein expression in the 7% CO_2_ (A) and 9% CO_2_ (B) groups at different time points (3, 6, and 9 days). (C) Protein level of ABCG2, p63, CK14, CK3, and OCT4 at 5%, 7%, and 9% CO_2_ at day 6 of differentiation. The undifferentiated hESCs were used as the control group.

The differentiated cells under 7% and 9% CO_2_ were used to analyze the changes in the ABCG2, p63, CK14, CK3, and OCT4 protein levels during the differentiation process because the expression of ABCG2, CK14, and CK3 could not be detected in the 5% CO_2_ group via western blotting. The results showed that the ABCG2, p63, and CK14 levels increased within 6 days of differentiation and declined at day 9. CK3 was detected in both groups at day 9; however, both bands were very weak (Fig 5A and 5B). Moreover, the expression of ABCG2, p63, and CK14 in the 7% CO_2_ group was higher than that in the other groups (Fig 5C).

### Investigation of non-corneal epithelial cell lineages and isolation of CEPCs derived from hESCs

**Fig 6:**
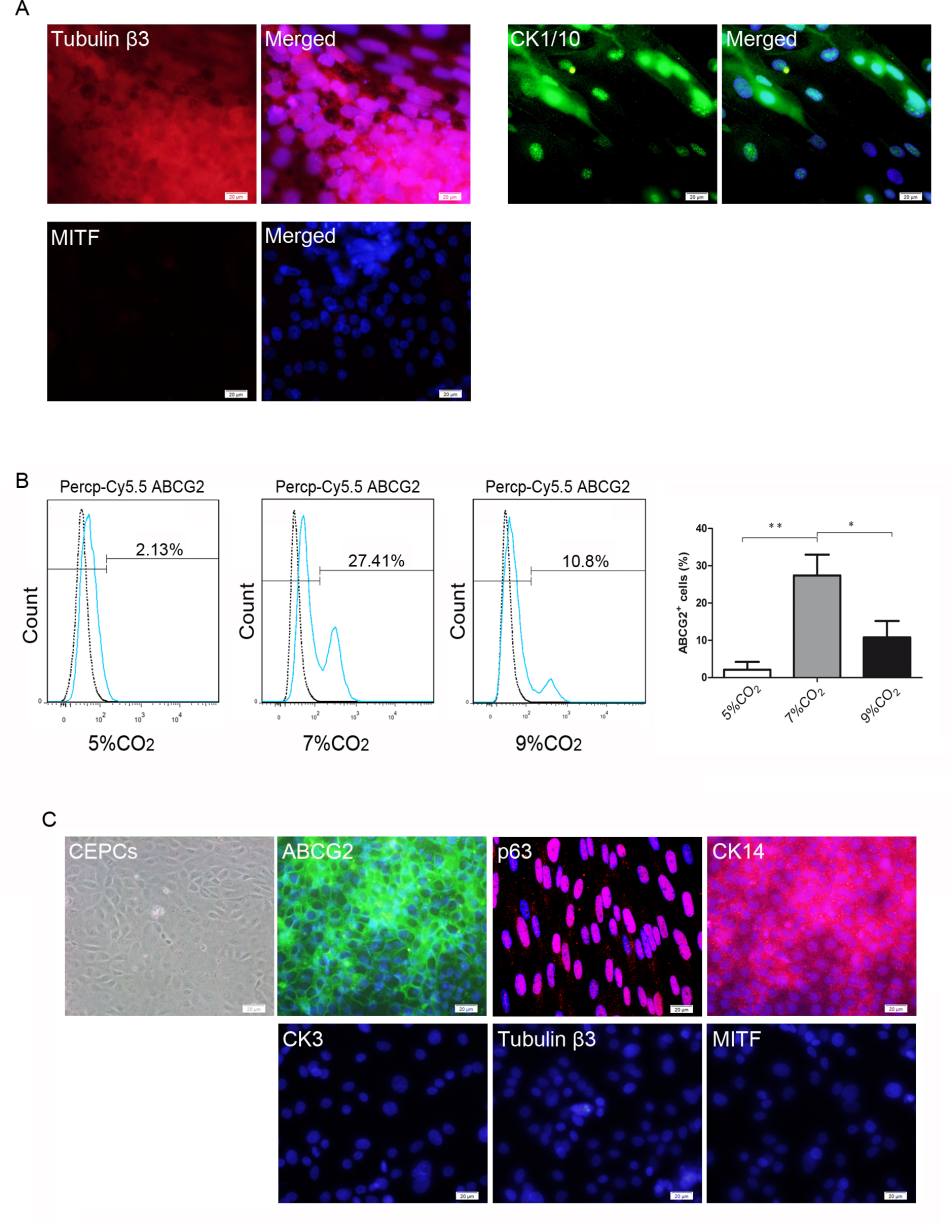
Immunostaining of non-corneal epithelial differentiation markers and isolation of CEPCs derived from hESCs by FACS. (A) Immunofluorescent staining of tubulin β3, CK1/10, and MITF. (B) FACS analysis of ABCG2-positive cells in different culture conditions at day 6 of differentiation. (C) Immunofluorescent staining of the sorted cells. ABCG2, p63, and CK14 were strongly expressed in the sorted cells. CK3, tubulin β3, and CK1/10 were not detected. Scale bar, 20 μm.

We determined that the differentiated cells could form identifiable different zones in larger clones, similar to previous report [26]; thus, the derivation of non-corneal epithelial cells was examined via immunofluorescence. The neural cell-specific marker tubulin β3, the epidermal marker K1/10, and the retinal pigment epithelial cell marker MITF were investigated (Fig 6A). The immunofluorescent signal of tubulin β3 was detected in the center of some larger clones; however, it could not be detected in the small clones. CK1/10 was also identified at the periphery of a small number of clones on the 12^th^ day of differentiation; however, MITF was not detected in the cultures. These findings indicate the presence of neural cell-like and epidermal cell-like cells in the differentiated cells; thus, the cell sorting was performed by FACS. ABCG2 is strictly confined to small clusters of basal cells in the limbal epithelium and may provide a suitable tool for the identification of human limbal stem cells (hLSCs) [43,44]; thus, ABCG2-positive cells were isolated by FACS. The data showed that the number of ABCG2-positive cells in the 7% CO_2_ group was significantly higher than that in the other groups, with an average of 27.41% of cells expressing ABCG2 at day 6 of differentiation (Fig 6B). The sorted cells were seeded on the plates coated with 2% growth factor reduced Matrigel as previously described. The expression of ABCG2, p63, and CK14, but not CK3, CK1/10, and tubulin β3, was detected in the cells via immunofluorescence (Fig 6C).

### Airlifting culture of CEPCs

**Fig 7:**
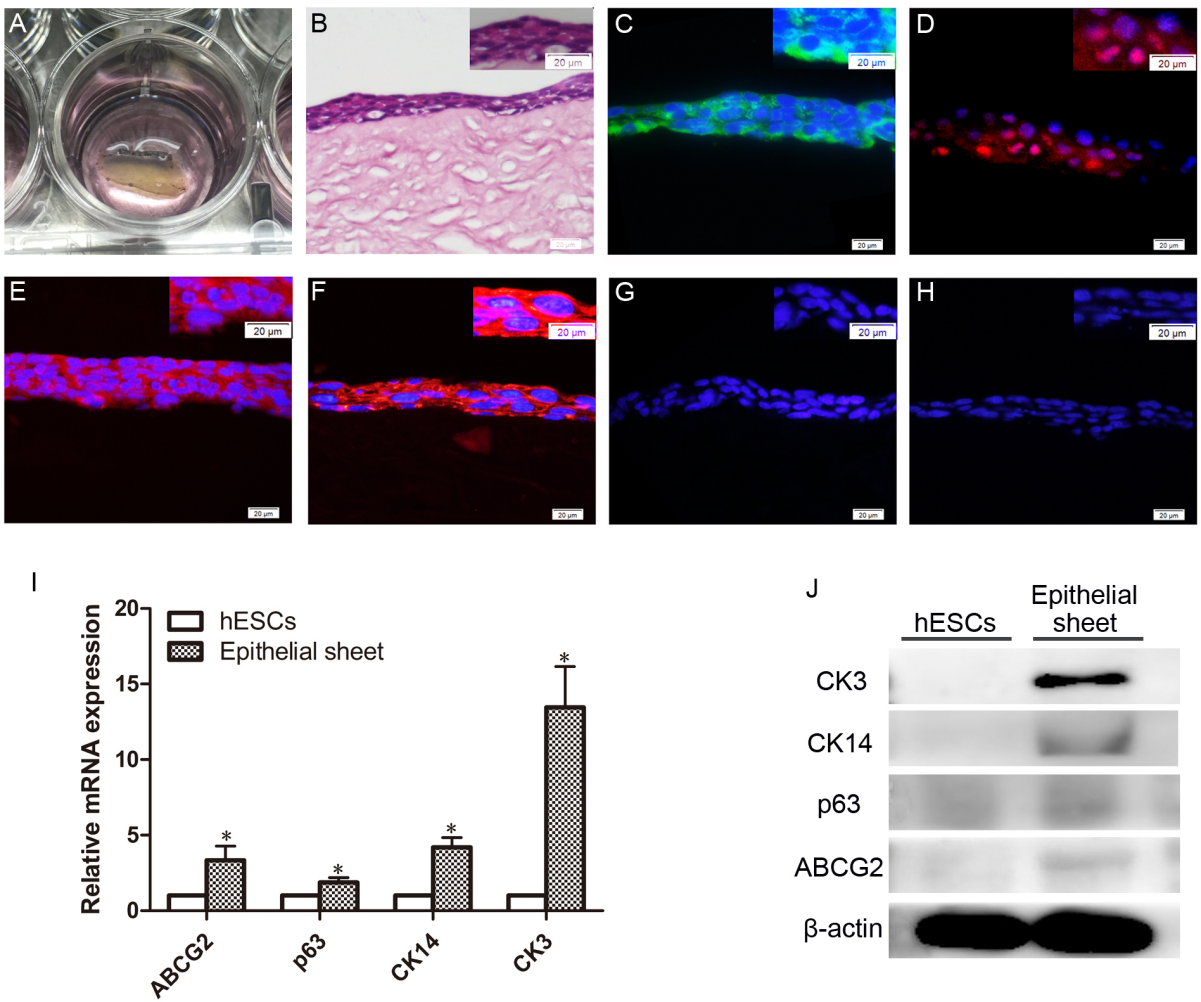
Characterization of epitheloid cell sheet on APLM. (A) Epitheloid cell sheets under airlifting culture. (B) HE staining of epitheloid cell sheet. (C-G) Immunofluorescent staining of ABCG2 (C), p63 (D), CK14 (E), CK3 (F), tubulin β3 (G), and CK1/10 (H) in the cells cultured on APLM. Scale bar, 20 μm. (I-J) Gene (I) and protein (J) expression of ABCG2, p63, CK14, and CK3 in epitheloid cell sheets. hESCs seeded on APLM were used as the control. Data are representative of three experiments and presented as the mean ± SD.*p < 0.05, **p < 0.01.

By airlifting culture on APLM, CEPCs formed stratified and closely arranged epithelioid cell sheets that consisted of three to four layers of elongated cells (Fig 7B). ABCG2, p63, CK14, and CK3 were detected in the epithelioid cell sheets via immunofluorescence; however, p63 was only expressed in some basal cells. CK1/10 and tubulin β3 were not identified in these cells by immunofluorescence (Fig 7C–7H). Quantitative RT-PCR analysis indicated that the ABCG2, p63, CK14, and CK3 levels in the epithelial sheets were significantly higher than those of the hESCs seeded on APLM (Fig 7I). However, p63 was not detected by western blotting, and the expression of ABCG2 and CK14 was very low in the epithelial sheets (Fig 7J). We suggest this may be caused by further differentiation of CEPCs into corneal epithelial cells on APLM.

## Discussion

In the present study, we established an effective and feasible strategy for the differentiation of CEPCs from hESCs *in vitro* under defined conditions. The sorted cells express the LSC markers ABCG2, p63, and CK14 and form stratified epithelioid cell sheets. These findings suggest that the differentiated cells have similar features to primary cultured hLSCs. Moreover, the protocol we developed provides a novel insight, other than mimicking the microenvironment of LSCs, into the differentiation of CEPCs.

During the differentiation process, we found the identifiable different zones in some large clones; however, most of the cells displayed an epithelial cell-like morphology and expressed corneal epithelial stem cell markers. FACS indicated that an average of 27.41% of the cells were ABCG2-positive. Therefore, cell sorting must be performed for the purification of CEPCs. CK14 and p63, but not CK3, CK1/10, and tubulin β3, were also detected in the sorted cells, which suggests that CEPCs of high purity could be attained in this protocol through cell sorting.

KSFM is a type of optimized medium used to support the growth and expansion of human keratinocytes, corneal epithelial cells, and LSCs [45–49]. Trans-membrane cadherins played an important role in the early-stage differentiation of hESCs, particularly in the differentiation of the ectoderm [50,51]. KSFM, mixed with DMEM/F12, increased its calcium concentration, which may facilitate the differentiation of ectodermal cells from hESCs. The expression of ABCG2, p63, CK14, CK3, CK1/10, and tubulin β3 in the differentiated cells demonstrated that hESCs cultured in KSFM-DMEM/F12 could differentiate into epithelial progenitor cells, including CEPCs. Furthermore, the feature of KSFM that facilitates epithelial or epithelial precursor cell growth may also be an important factor in the differentiation of CECs from hESCs. However, the explicit mechanisms require intensive study.

The expression of ABCG2, p63, and CK14 decreased at day 9 compared with day 6 of differentiation, whereas the CK3 levels started to increase in our studies, which suggested that this culture system could not serve as the niche in the long-term maintenance of LSC properties, and corneal epithelial differentiation had progressed further during the differentiation. These findings also indicated that the 6^th^ day of differentiation may be a suitable time to perform cell sorting of CEPCs from cultures in this differentiation model. The expression of CEPC markers, particularly ABCG2 and CK14, was substantially higher in the differentiated cells under 7% CO_2_ than that under 5% CO_2_, which indicates that slightly elevated CO_2_ was conductive to the differentiation of CEPCs from hESCs. However, the involved mechanism remained unclear. Previous studies have indicated that elevated CO_2_ enhances embryo development by its action as a weak acid to maintain the appropriate intracellular pH [16]. Moreover, hypercapnia caused nuclear translocation of IKKα, and IKKα is implicated in the differentiation of epithelial cells [52,53]. We speculate these factors may play roles in the differentiation of CEPCs from hESCs. The invasive ability of colon cancer cells increased under hypercapnic conditions [54], and elevated levels of CO_2_ are favored for the enhanced proliferation of bone marrow (BM) progenitor cells [55]. Similar to previous reports, in this study, we determined that the cell density in the cultures under 7% and 9% CO_2_ was higher than 5% CO_2_ on the 9^th^ and 12^th^ days of differentiation, which indicated that slightly elevated levels of CO_2_ may promote the proliferation of differentiated hESCs.

APLM and the limbal microvascular net play important roles as components of the limbal epithelial stem cell niche in the avoidance of the differentiation of limbal epithelial stem cells [24,56]. Our previous studies [35,36] have indicated that acellular porcine corneal matrix developed by sodium dodecyl sulfate (SDS) maintained the intact basement membrane and possessed good biocompatibility. The cells could form three to four layers of epithelioid cells on APLM by airlifting culture, and CK3 was detected in most cells; however, the expression of corneal epithelial stem cell markers, ABCG2 and p63, was very low. These data demonstrated that APLM without the limbal microvascular net could not maintain the limbal epithelial stem cell stemness. CEPCs derived from hESCs could generate corneal epithelial-like cells, which suggests that the cells seeded in a suitable corneal epithelial stem cell niche may be used in corneal tissue engineering or the treatment of LSCD. However, the derivation of ES cells from the human embryo raises ethical questions, in part because the predominant methods used require destruction of the embryo. Compared with ESCs, induced pluripotent stem (iPS) cells avoid ethical issues and may be propagated as autologous cells without immunological rejection after transplantation [57]. Previous studies have indicated that iPS cells functionally equivalent to ESCs can generate cell types from each of the three embryonic germ layers: the endoderm, mesoderm and ectoderm [58,59]. We conclude that the differentiation method in this study may also be suitable for the derivation of epithelial lineage or corneal epithelial lineage from iPS cells.

## Conclusions

In summary, we developed a novel protocol, which differs from mimicking the CEPC niche, to differentiate hESCs into CEPCs, and the differentiated cells exhibited similar morphological features, cell markers, and differentiation capacity as hLSCs. This differentiation model may provide a new step toward fully defined and xeno-free differentiation protocols to produce CEPCs for investigations of basic biology. Moreover, with the improvement of the cell sorting technique and the emergence of CEPC-specific markers, these differentiated cells hold substantial promise in regenerative medicine.

## Supporting information

S1 File The data of qRT-PCR. (ZIP)

S2 File The FACS analysis results of ABCG2-positive cells. (ZIP)
